# 986. Clinical Utility of 16S rRNA Sequencing and Impact on Outcomes in Adults with Suspected Infection

**DOI:** 10.1093/ofid/ofad500.041

**Published:** 2023-11-27

**Authors:** Kaitlyn Weinert-Stein, Leila S Hojat, Sree Sarah Cherian, Peter Paul Lim, Ankita P Desai, Mackenzie Cater, Ana Khazan, Reem Azem, LeAnne Moore, Lisa M Stempak

**Affiliations:** University Hospitals Cleveland Medical Center, Cleveland, OH; Case Western Reserve University/ University Hospitals Cleveland Medical Center, Cleveland, Ohio; University Hospitals - Cleveland Medical Center, Cleveland, Ohio; Avera McKennan University Health Center, Sioux Falls, South Dakota; University Hospitals Rainbow Babies and Children's Hospitals, Cleveland, Ohio; Case Western Reserve University/University Hospitals, Cleveland Heights, Ohio; Case Western Reserve/University Hospitals, Cleveland, Ohio; University Hospitals Cleveland Medical Center, Cleveland, OH; Rainbow Babies and Children's Hospital, Cleveland, Ohio; University Hospitals, Lyndhurst, Ohio

## Abstract

**Background:**

Targeted antimicrobial regimens rely on prompt isolation and identification of the pathogen. When conventional gold standard culture-based methods fail to identify a pathogen, broad range PCR and next-generation 16S rRNA sequencing has emerged as a clinically useful and increasingly frequent diagnostic tool. However, its impact in clinical decision making and effect on outcomes, particularly with negative results, remains poorly elucidated. This study aims to evaluate the clinical impact of 16S rRNA in adult infections.

**Methods:**

A retrospective analysis was performed on clinical specimens analyzed by 16S rRNA from August 2016 to December 2021 in our institution. Electronic medical records were reviewed to determine if 16S rRNA results had clinical utility, defined as prompting a change or stop of therapy or confirming the prescribed antimicrobial regimen. Antimicrobial-related events, readmission and infection-related mortality at 90 days were the outcomes of interest and were compared between groups using the Fisher's exact test.

**Results:**

Three hundred fifty-nine samples were included in the analysis. Clinical utility was identified in 108 (30.1%) specimens including 45 (41.7%) with negative 16S rRNA results (Figure 1). The most common pathogens identified were skin flora (19.4%) and *Streptococci spp.* (13.9%). Body fluid specimens yielded the most clinical utility (62.5%) followed by cranial samples (60%) and valve/endovascular graft samples (52%) (Figure 2). Readmissions were significantly lower in the clinical utility group compared to the no clinical utility group (10.5% vs 19.8%, p=0.04, 95% confidence interval 0.21-0.99) (Table 1). Mortality and adverse events were not statistically different between the two groups.

**Figure 1.**
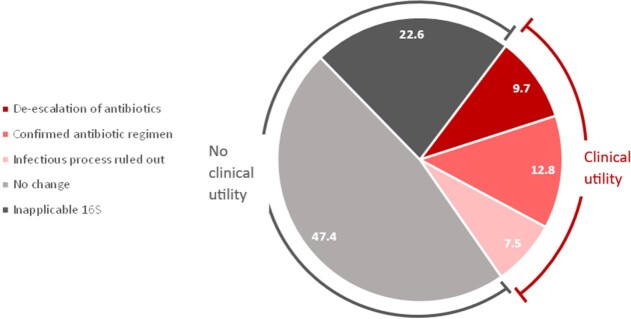
Proportion of clinical specimens stratified by clinical impact of 16S rRNA sequencing. De-escalation of antibiotics = narrowing coverage or shortening course. Confirmation of antibiotic regimen = confirming current coverage, escalation of coverage, or lengthening course. Infectious process ruled out = low baseline suspicion for infection. No change = negative 16S rRNA result or those lost to follow-up (true negative utility). Inapplicable 16S rRNA = positive culture result before 16S rRNA result or intended therapy less than or equal to 14 days.

**Figure 2.**
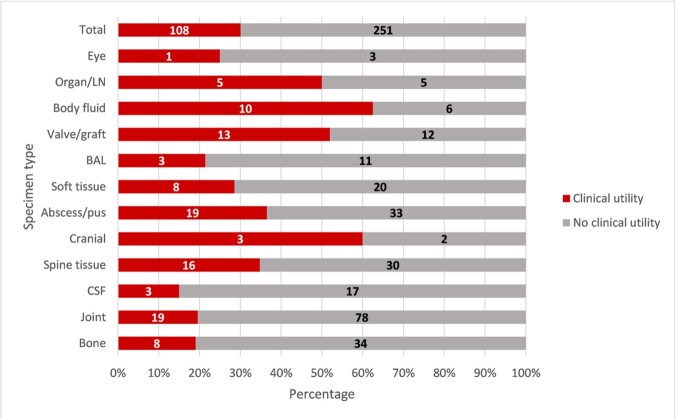
The clinical utility of 16S rRNA sequencing results by clinical specimen type. Body fluid included pericardial fluid, peritoneal fluid, middles ear fluid, and cyst/seroma fluid. LN: lymph node, BAL: bronchoalveolar lavage, CSF: cerebrospinal fluid.

**Table 1.**
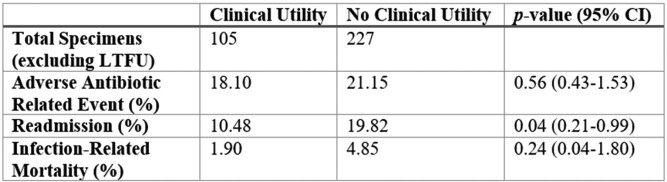
Comparison of outcomes between clinical utility and no clinical utility groups. LTFU: lost to follow-up, CI: confidence interval.

**Conclusion:**

Both positive and negative 16S rRNA results have clinical utility in our study with body fluid specimens having the highest clinical utility. Readmissions were statistically lower in cases with clinical utility, while adverse events and mortality were numerically lower but not statistically significant. To our knowledge, this is the first study to demonstrate the impact of negative 16S rRNA results in adult infections, and further studies are needed to determine the most effective application for patient care.

**Disclosures:**

**Lisa M, Stempak, MD**, Cytovale Inc: Advisor/Consultant

